# WNT/β-Catenin Signaling in Vertebrate Eye Development

**DOI:** 10.3389/fcell.2016.00138

**Published:** 2016-11-30

**Authors:** Naoko Fujimura

**Affiliations:** Laboratory of Eye Biology, BIOCEV Division, Institute of Molecular GeneticsPrague, Czechia

**Keywords:** retina, WNT, β-catenin, development, differentiation

## Abstract

The vertebrate eye is a highly specialized sensory organ, which is derived from the anterior neural plate, head surface ectoderm, and neural crest-derived mesenchyme. The single central eye field, generated from the anterior neural plate, divides to give rise to the optic vesicle, which evaginates toward the head surface ectoderm. Subsequently, the surface ectoderm, in conjunction with the optic vesicle invaginates to form the lens vesicle and double-layered optic cup, respectively. This complex process is controlled by transcription factors and several intracellular and extracellular signaling pathways including WNT/β-catenin signaling. This signaling pathway plays an essential role in multiple developmental processes and has a profound effect on cell proliferation and cell fate determination. During eye development, the activity of WNT/β-catenin signaling is tightly controlled. Faulty regulation of WNT/β-catenin signaling results in multiple ocular malformations due to defects in the process of cell fate determination and differentiation. This mini-review summarizes recent findings on the role of WNT/β-catenin signaling in eye development. Whilst this mini-review focuses on loss-of-function and gain-of-function mutants of WNT/β-catenin signaling components, it also highlights some important aspects of β-catenin-independent WNT signaling in the eye development at later stages.

## Overview of eye development in mice

**Figure 1:**
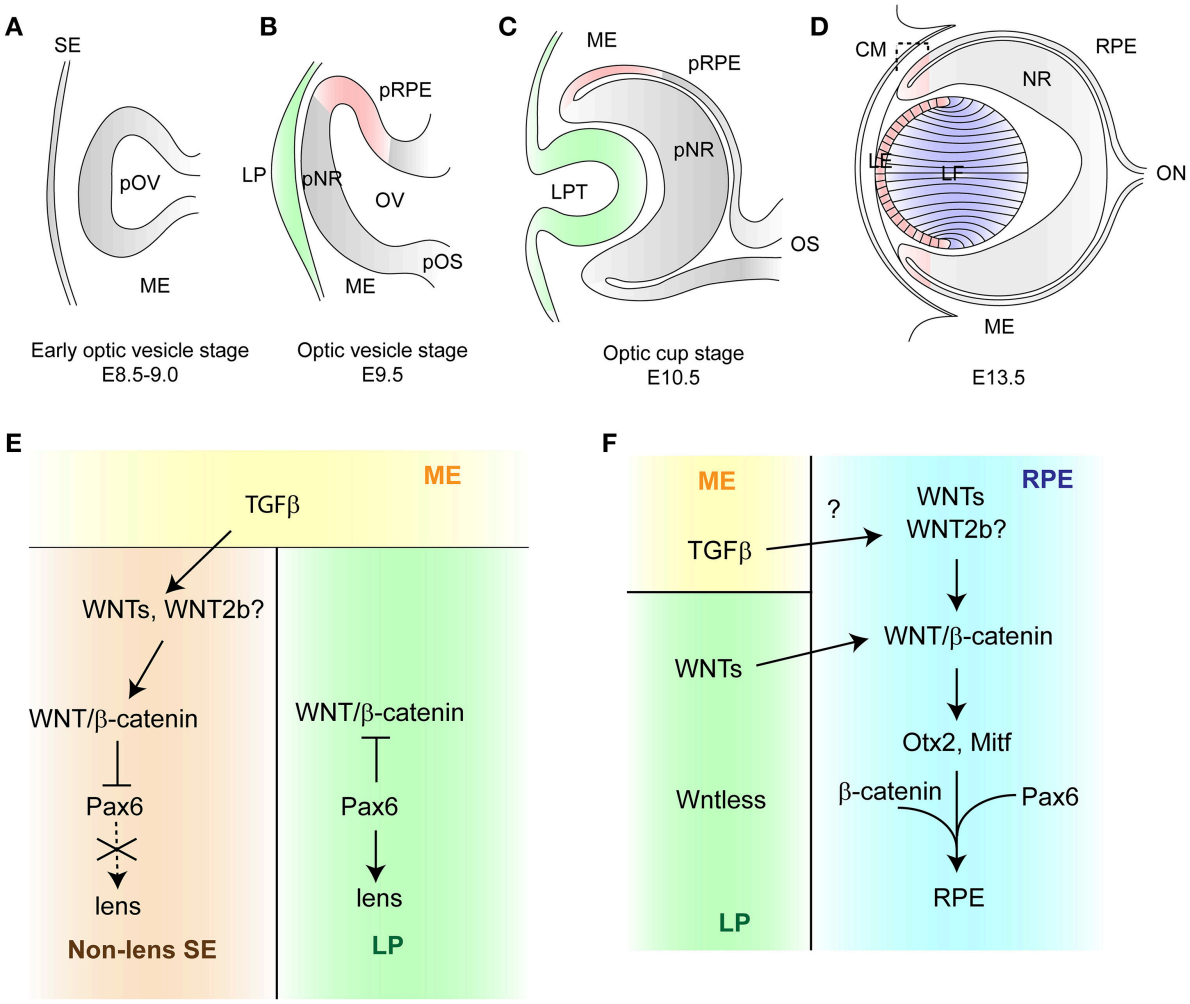
**Schematic diagram of vertebrate eye development (A)**. The early optic vesicle stage (E8.5–9.0). The presumptive optic vesicle envaginates toward the head surface ectoderm through the mesenchyme. **(B)** The optic vesicle stage (E9.5). As the optic vesicle comes into contact with the head surface ectoderm, it becomes partitioned into three domains: a dorsal, a distal and a proximal domain, which give rise to the retinal pigment epithelium, the neural retina and the optic stalk, respectively. The head surface ectoderm thickens to form the lens placode. **(C)** The optic cup stage (E10.5). The optic vesicle invaginates in coordination with the lens placode to form the optic cup and the lens pit. **(D)** The closure of the lens vesicle (E13.5). The cells located at the posterior lens vesicle elongate anteriorly to fill the cavity and differentiate as primary lens fiber cells. The cells in the anterior part of lens vesicle give rise to lens epithelial cells which migrate posteriorly to the equator and differentiate as secondary lens fiber cells. Pink color represents the region where the activity of WNT/β-catenin signaling is active, green shows the source of WNTs, blue indicates the region where WNT/PCP signaling is active. **(E, F)** Schematic representation of WNT/β-catenin signaling in the early lens development and in the RPE development, respectively. E. The periocular mesenchyme secretes TGFβ, which signals to the non-lens surface ectoderm. *WNT2b* is induced by TGFβ and activates WNT/β-catenin signaling in order to suppress the lens fate by repressing expression of *Pax6*. In the lens placode, WNT/β-catenin is inhibited by *Pax6* which initiates lens development. **(F)** The surface ectoderm secretes WNTs which activate WNT/β-catenin signaling in the RPE. This signaling induces expression of *Otx2* and *Mitf* which in cooperation with *Pax6* control the RPE developments.

During gastrulation, the eye field, a group of the retinal precursor cells, is specified within the anterior neural plate. At this stage, these cells are anteriorly and laterally surrounded by the telencephalic progenitor cells. Subsequently, the eye field is divided into two lateral parts, which extend toward the surface ectoderm and give rise to the optic vesicle (Figure 1A; Inoue et al., 2000; Cavodeassi and Houart, 2012; Heavner and Pevny, 2012). The head surface ectoderm thickens to give rise to the lens placode while the optic vesicle subdivides into three parts, namely the presumptive retinal pigment epithelium (RPE), the presumptive neural retina, and the presumptive optic stalk (Figure 1B). The optic vesicle subsequently invaginates together with the lens placode to form the double-layered optic cup (Figure 1C). The inner part of the optic cup gives rise to the neural retina, meanwhile the outer layer forms the RPE. The ciliary margin (peripheral part of the optic cup) develops to generate the iris and the ciliary body. The lens placodes progresses to form a hollow lens vesicle. Cells in the posterior region differentiate as primary lens fiber cells and elongate to fill the cavity, while the cells in the anterior region become proliferative lens epithelial cells (Figure 1D; Fuhrmann, 2008; Cvekl and Ashery-Padan, 2014; Fuhrmann et al., 2014). The retinal vessels arise from the optic nerve head shortly after birth and extend radically to the retinal periphery in the superficial retina. The vasculature then sprouts ventrally to form the deep vascular layer (Gariano and Gardner, 2005).

## WNT signaling

WNTs can couple to various receptors and trigger different downstream signaling cascades including the non-canonical WNT/planar cell polarity (PCP), WNT/Ca^2+^, and the canonical WNT/β-catenin signaling pathway, the focus of this review. WNT/β-catenin signaling is initiated by binding of the WNTs to the Frizzled/LRP5/6 receptor complex, which leads to the accumulation of β-catenin and nuclear translocation. In the nucleus, β-catenin interacts with the TCF/LEF family of transcription factors and regulates their target genes. In the absence of WNTs, β-catenin is phosphorylated by a “destruction complex” composed of multiple proteins, including AXIN2 and GSK3β, and targeted for degradation (Loh et al., 2016). In addition to its critical role as a transcriptional co-activator, β-catenin acts as a central component of the adherens junction by forming a link between cadherins and the actin cytoskeleton (Heuberger and Birchmeier, 2010). WNT/PCP signaling does not use β-catenin, but activates the Rho family GTPases and JNK pathway, which results in changes in cytoskeleton and cell polarity (Loh et al., 2016). WNT signaling is modulated by a number of WNT-sequestering proteins, such as DKKs and SFRPs, which prevent ligand-receptor interactions (Cruciat and Niehrs, 2013).

## The lens

WNT signaling plays essential roles in eye organogenesis (Fuhrmann, 2008). During lens development, WNT/β-catenin signaling is active in the periocular surface ectoderm and lens epithelium (Stump et al., 2003; Smith et al., 2005; Kreslova et al., 2007; Machon et al., 2010; Carpenter et al., 2015). Conditional deletion of β*-catenin* in the presumptive lens placode and surrounding head surface ectoderm results in abnormal lens morphogenesis due to cell-cell adhesion defects. Conversely, the lens induction in the β*-catenin* loss-of-function mutant is not affected as expression of lens-specific markers is maintained (Smith et al., 2005). Consistently, a null mutation in *Lrp6*, which is expressed throughout the eye at the optic vesicle stage, does not have a profound effect on the lens induction (Stump et al., 2003; Smith et al., 2005). Interestingly, ectopic lentoid bodies are formed in the periocular surface ectoderm, where WNT/β-catenin signaling is inactivated in β*-catenin*-deficient mutants. Although the adherens junction is disrupted, ectopic lentoid bodies are not observed in the *E-cadherin*/*N-cadherin* or *Scribs* conditional knockout mice generated using the same Cre line (Pontoriero et al., 2009; Yamben et al., 2013). Thus, formation of ectopic lentoid bodies is mediated by the inactivation of WNT/β-catenin signaling rather than by cell-cell adhesion defects. In addition, ectopic activation of WNT/β-catenin signaling by expression of constitutively active β-catenin leads to inhibition of the lens formation (Smith et al., 2005; Machon et al., 2010). Taken together, WNT/β-catenin signaling is not required for the lens fate determination, however it inhibits the lens formation and appears to suppress the lens fate in the periocular ectoderm. The precise regulation of WNT/β-catenin signaling is required to ensure the correct patterning of the ocular tissue.

WNT/β-catenin signaling is regulated by TGFβ signaling and *Pax6* in the surface ectoderm at the optic vesicle stage (Figure 1E). The migrating neural crest cells inhibit the lens specification, while their ablation results in ectopic lens formation (Bailey et al., 2006). In chick embryos, the neural crest cells secrete multiple TGFβs which activate WNT/β-catenin signaling by inducing *WNT2b* in the adjacent non-lens ectoderm. The lens fate in presumptive lens ectoderm explants can be suppressed by the neural crest, constitutively active β-catenin, as well as TGFβ. Interestingly, the expression of lens markers is restored when these explants are cultured with TGFβ and WNT-sequestering protein FZD8-CRD, a truncated and soluble form of the WNT receptor. This indicates that lens suppression by the neural crest-derived TGFβ is dependent on WNT/β-catenin signaling (Grocott et al., 2011). *WNT2b* null mice display no ocular defects and multiple WNTs are expressed in the surface ectoderm, therefore additional WNTs are required for the process in mice (Tsukiyama and Yamaguchi, 2012; Carpenter et al., 2015).

*Pax6* is expressed in the presumptive lens placode and *Pax6* null mutation results in failure of the lens formation (Hill et al., 1991; Grindley et al., 1995). It has been shown that *Pax6* regulates the expression of *Sfrp2*, and *Dkk1*. In *Pax6-*deficient presumptive lens placode, *Sfrp2* is down-regulated and WNT/β-catenin signaling is ectopically activated (Machon et al., 2010). However, it is unlikely that *Sfrp2* acts as a downstream effector as lens induction is not affected in the *Sfrp1*^−/−^*; Sfrp2*^−/−^ mice (Sugiyama et al., 2013). On the other hand, the role of *Dkk1* in the lens induction remains elusive as *Dkk1* null embryos lack the anterior head structure including the eyes (Mukhopadhyay et al., 2001). Interestingly, PAX6 ChIP sequencing using human neuroectodermal cells has shown that PAX6 binds to a variety of genes, which regulate WNT signaling (Bhinge et al., 2014). Further studies are necessary to understand how *Pax6* counteracts WNT/β-catenin signaling.

At later stages of development, WNT/β-catenin signaling is required for the formation and maintenance of the lens epithelium (Stump et al., 2003; Cain et al., 2008; Martinez et al., 2009). Interestingly, WNT/β-catenin signaling is reduced in the lens epithelium of the *Sfrp1*^−/−^*; Sfrp2*^−/−^ embryos (Sugiyama et al., 2013). SFRP1/2 are primarily characterized as WNT-sequestering proteins, however they can activate WNT/β-catenin signaling by facilitating the diffusion of WNTs or suppressing WNT/PCP pathway which can antagonize WNT/β-catenin signaling (Satoh et al., 2008; Mii and Taira, 2009). Additionally, *Sfrp1/2* can also inhibit BMP and Notch signaling, which are required for lens development, thus mis-regulation of these signaling pathways might also be responsible for the defects in the *Sfrp1/2-*deficient lens (Misra and Matise, 2010; Esteve et al., 2011a).

Although WNT/β-catenin signaling is not required for the lens fiber development, there are indications that the alignment and orientation of lens fiber cells are dependent on the WNT/PCP signaling pathways (Chen et al., 2008; Sugiyama et al., 2010, 2011). In the lens overexpressing *Sfrp2*, the fiber orientation is severely disrupted and expression of components of the WNT/PCP pathway is down-regulated (Chen et al., 2008; Sugiyama et al., 2010). WNT5, which activates the PCP pathway is secreted from the lens epithelium and WNT5 promotes the directed behavior of lens fiber cells in the lens explants (Dawes et al., 2014).

## The RPE

Signals from neighboring tissues are crucial for the accurate specification of the neural retina and the RPE within the optic vesicle. The dorsal optic vesicle receives signals from the extraocular mesenchyme and the head surface ectoderm to differentiate into the RPE (Fuhrmann et al., 2000; Martínez-Morales et al., 2004; Bharti et al., 2006; Steinfeld et al., 2013; Carpenter et al., 2015). During retinal development, WNT/β-catenin signaling is active in the dorsal optic vesicle which gives rise to presumptive RPE at the optic vesicle stage and is subsequently restricted to the peripheral RPE (Liu et al., 2006; Fujimura et al., 2009; Westenskow et al., 2009; Hägglund et al., 2013). The RPE transdifferentiates into the neural retina in the β*-catenin*-deficient RPE at the optic cup stage, as evidenced by loss of the RPE markers *Mitf* and *Otx2* and by the ectopic expression of neural retinal markers, such as *Chx10* and *Rax* (Fujimura et al., 2009; Westenskow et al., 2009; Hägglund et al., 2013). The β-catenin-deficient RPE preserves intact adherens junctions at the optic cup stage, although cell-cell adhesion is disrupted at later stages (Fujimura et al., 2009; Westenskow et al., 2009). Interestingly, γ-catenin, a paralog of β-catenin, can substitute β-catenin in cell adhesion complexes in various developmental contexts (Huelsken et al., 2000; Posthaus et al., 2002; Zhou et al., 2007). The lack of β-catenin in the adherens junctions might be compensated by γ-catenin as evidenced by the presence of γ-catenin in the β-catenin-deficient RPE at the optic cup stage. Thus, the transdifferentiation is probably caused by loss of WNT/β-catenin signaling (Fujimura et al., 2009). A similar phenomenon is observed in the optic cup derived from the mouse embryonic stem cell aggregates *in vitro* (Eiraku et al., 2011). Treatment with a WNT secretion inhibitor reduces the number of the RPE cells, while WNT3a promotes the RPE differentiation and suppresses the neural retina generation (Eiraku et al., 2011). Interestingly, ectopic activation of WNT/β-catenin signaling in the entire RPE also results in disruption of the RPE patterning. The peripheral RPE remains normal, while the central part, in which WNT/β-catenin signaling is ectopically active, loses expression of the RPE markers. In contrast to β*-catenin*-deficient mutants, the RPE is not transdifferentiated to the neural retina (Fujimura et al., 2009). Thus, the activity of WNT/β-catenin signaling is spatially and temporally regulated during the RPE development.

WNT/β-catenin signaling regulates RPE development in cooperation with *Mitf*, *Otx2*, and *Pax6* (Figure 1F). Expression of *Mitf* and *Otx2* is directly regulated by WNT/β-catenin signaling (Fujimura et al., 2009; Westenskow et al., 2009). Furthermore, ectopic expression of both *Otx2* and β*-catenin* in the presumptive chick neural retina promotes the RPE fate while the ectopic expression of *Otx2* or β*-catenin* alone is not sufficient. Therefore, β*-catenin*, together with *Otx2*, induces a change in cell fate from retinal progenitor cells to the presumptive RPE (Westenskow et al., 2010). Furthermore, β-catenin directly interacts with MITF and promotes *Mitf* -mediated transcription (Schepsky et al., 2006). A recent study has shown that PAX6 acts in synergy with β-catenin and MITF to activate the promoters of melanogenic genes *Tyr* and *Trp-1* (Fujimura et al., 2015).

Although the identity of the specific WNTs involved in RPE development remains elusive, a recent study has shown that WNTs from the surface ectoderm are necessary for this process (Carpenter et al., 2015). During early eye development, the WNT transporter *Wntless* is expressed in the presumptive lens placode, the periocular surface ectoderm, the periocular mesenchyme at the optic vesicle stage, and it is also detected in the peripheral retina and the RPE at later stages (Carpenter et al., 2015). Conditional deletion of *Wntless* in the presumptive lens leads to inactivation of WNT/β-catenin signaling in the peripheral retina and periocular mesenchyme (Carpenter et al., 2015). Moreover, the number of RPE cells is reduced in *Wntless*-deficient mice (Carpenter et al., 2015). Despite the presence of multiple WNTs and *Wntless* in the periocular mesenchyme, conditional inactivation of *Wntless* in the periocular mesenchyme and RPE does not affect the eye development or the activity of WNT/β-catenin signaling (Carpenter et al., 2015). It remains elusive how WNTs disperse from the periocular mesenchyme to the WNT-responsive tissue in the optic cup. There are, however, indications that heparan sulfate proteoglycans (HSPG) are involved in the distribution of WNTs within the eye. HSPGs are located on the cell surface and in the extracellular matrix and have been implicated in a number of signaling pathways including WNT (Sarrazin et al., 2011). In the context of WNT signaling transduction, HSPGs play an essential role in organizing the extracellular distribution of WNTs and they maintain the activity of WNTs by preventing their aggregation in the extracellular environment (Fuerer et al., 2010; Matsuo and Kimura-Yoshida, 2014). Interestingly, conditional deletion of *Ext1*, a key HSPG synthetic enzyme, in the periocular mesenchyme leads to severe ocular malformations including the defects in the peripheral RPE development (Iwao et al., 2010). It has not been shown whether WNT/β-catenin signaling is affected in the peripheral optic cup of the *Ext1-*deficient mice, however *Ext1* is required for the activation of the WNT11/β-catenin pathway in *Xenopus* embryos (Tao et al., 2005). Thus, HSPG in the periocular mesenchyme might mediate the distribution of WNTs from the surface ectoderm.

## The ciliary margin

WNT/β-catenin signaling is active in the developing ciliary margin or peripheral retina, but it is inactive in the central retina (Liu et al., 2003, 2007; Cho and Cepko, 2006). Several WNT signaling members, such as *WNT2b, Frizzled-4* (*FZD*_4_), and *Lef1* are expressed in the ciliary margin (Trimarchi et al., 2009). Overexpression of a constitutively active form of β-catenin leads to the expansion of the ciliary margin at the expense of the central retina (Cho and Cepko, 2006; Liu et al., 2007; Trimarchi et al., 2009). In addition, *Axin2* null embryos display multiple ocular phenotypes including expansion of the ciliary margin (Alldredge and Fuhrmann, 2016).

Several studies indicate that WNT/β-catenin signaling activity in the peripheral retina is controlled by *Sfrp1/2, Foxg1*, and *Sox2* (Matsushima et al., 2011; Esteve et al., 2011b; Fotaki et al., 2013). As mentioned above, it has been suggested that WNT-sequestering proteins SFRP1/2 can activate WNT/β-catenin signaling (Bovolenta et al., 2008). In the *Sfrp1*^−/−^*; Sfrp2*^−/−^ embryos, this signaling is inactive in the peripheral retina, which displays neural retinal characteristics (Esteve et al., 2011b). Conversely, restriction of WNT/β-catenin signaling to the ciliary margin has been shown to be mediated by *Foxg1* and *Sox2* (Matsushima et al., 2011; Fotaki et al., 2013). In *Foxg1*−or *Sox2-*deficient retina, WNT/β-catenin signaling are up-regulated in the peripheral retina and the ciliary margin expands at the expense of the neural retina (Matsushima et al., 2011; Fotaki et al., 2013). *foxg1* suppresses WNT/β-catenin signaling by directly repressing the transcription of WNTs in the forebrain of zebrafish (Matsushima et al., 2011). SOX2 interferes with WNT/β-catenin signaling by binding β-catenin in the osteoblast lineage (Seo et al., 2011). Taken together, it is likely that multiple mechanisms control the activity of WNT/β-catenin signaling in the ciliary margin.

## The dorso-ventral patterning in the optic cup

In addition to the correct patterning of the lens and the RPE development, WNT/β-catenin signaling is required for the maintenance of the dorsal retinal identity (Veien et al., 2008; Zhou et al., 2008; Hägglund et al., 2013). Conditional inactivation of β*-catenin* in the early optic cup results in the down-regulation of dorsal retinal markers, such as *Bmp4* and expansion of the ventral retinal markers, such as *Vax2* (Hägglund et al., 2013). Similarly, loss of *Lrp6* causes dorso-ventral patterning defects in the neural retina (Zhou et al., 2008). Consistently, the expression of dorsal retinal markers are attenuated in a transgenic fish which overexpresses *dkk1* or dominant-repressor form of *tcf3.* This phenotype is rescued by LiCl, which promotes the accumulation of cytoplasmic β-catenin by inhibiting GSK3β (Veien et al., 2008). Thus, the role of WNT/β-catenin signaling in the dorso-ventral patterning within the retina seems to be evolutionarily conserved.

## The retinal vascular system

WNT/β-catenin signaling plays an essential role in the retinal vascular development. In genetic disorders, such as Norrie disease and Familial Exudative Vitreoretinopathy, retinal hypovascularization is caused by loss-of-function mutations in the *Norrin disease protein (Norrin), FZD*_4_, or *LRP5* genes. Norrin contains separate binding sites for FZD_4_ and for LRP5 (Ke et al., 2013). Activation of FZD_4_/β-catenin signaling by Norrin requires the presence of either LRP5 or LRP6 (Ye et al., 2009). Although *Lrp5* can compensate for the loss of *Lrp6* (and vice versa) in the postnatal brain vasculature, *Lrp5* plays a major role and *Lrp6* plays a minor role in the retinal vascularization (Zhou et al., 2014; Huang et al., 2016). Norrin secreted from Müller glial cells binds to FZD_4_ in the endothelial cells and regulates retinal vascular development (Xu et al., 2004; Junge et al., 2009; Ye et al., 2009; Wang et al., 2012). The retinal vascular defects caused by ablation of *Norrin* are rescued by stabilizing β-catenin, while ectopic expression of dominant negative *Tcf4* in the endothelial cells mimics the phenotype. This indicates that Norrin/FZD_4_ signaling acts via β-catenin signaling (Zhou et al., 2014). In addition, WNT/β-catenin signaling in the retinal vascular system is regulated by the EST transcription factor *Erg*, which plays a critical role in vascular development and angiogenesis (Birdsey et al., 2015). *Erg* controls WNT/β-catenin signaling by promoting β-catenin stability and regulating transcription of *FZD*_4_ (Birdsey et al., 2015).

β-catenin-independent WNT signaling pathway is also required for the retinal vascular system development (Stefater et al., 2011; Korn et al., 2014; Franco et al., 2016). The endothelial cells express preferentially non-canonical WNTs, such as *WNT5a* and *WNT11*. Conditional deletion of *Wntless* or *WNT5a* in the endothelial cells leads to significant decrease in vascular density due to excessive vessel regression (Korn et al., 2014; Franco et al., 2016).

## Conclusion

The activity of WNT/β-catenin signaling is tightly regulated during eye development and mis-regulation of the signaling results in multiple ocular malformations due to defects in the process of cell fate determination and differentiation. Studies of conditional knockout mice of various members of the WNT/β-catenin signaling pathway indicate that WNT/β-catenin signaling is essential for eye development by controlling the correct patterning of the ocular tissue, promoting the differentiation of the retinal pigment epithelium, controlling the morphogenesis of the optic cup, and maintaining the dorsal retinal identity. Further research is necessary to clarify the mechanisms through which WNT/β-catenin signaling integrates into the genetic regulatory networks controlling the eye development in the vertebrate.

## Author contributions

The author confirms being the sole contributor of this work and approved it for publication.

## Funding

This work was supported by the Ministry of Education, Youth and Sports of CR within the LQ1604 National Sustainability Program II (Project BIOCEV-FAR).

### Conflict of interest statement

The author declares that the research was conducted in the absence of any commercial or financial relationships that could be construed as a potential conflict of interest.

## Abbreviations

pOV: presumptive optic vesicle
OV: optic vesicle
SE: head surface ectoderm
ME: extraocular mesenchyme
pRPE: presumptive retinal pigment epithelium
pNR: presumptive neural retina
pOS: presumptive optic stalk
LP: lens placode
RPE: retinal pigment epithelium
LPT: lens pit
OS: optic stalk
CM: ciliary margin
LE: lens epithelium
ON: optic nerve.
